# Macrophage polarization and metabolic reprogramming in abdominal aortic aneurysm

**DOI:** 10.1002/iid3.1268

**Published:** 2024-11-12

**Authors:** Ningxin Hou, Hongmin Zhou, Jun Li, Xiaoxing Xiong, Hongping Deng, Sizheng Xiong

**Affiliations:** ^1^ Division of Cardiovascular Surgery, Tongji Hospital, Tongji Medical College Huazhong University of Science and Technology Wuhan China; ^2^ Department of Neurosurgery Renmin Hospital of Wuhan University Wuhan China; ^3^ Department of Vascular Surgery Renmin Hospital of Wuhan University Wuhan China

**Keywords:** abdominal aortic aneurysm, inflammation, macrophage polarization, metabolic reprogramming, therapeutic targets

## Abstract

**Background:**

Abdominal aortic aneurysm (AAA) is a macrovascular disease with high morbidity and mortality in the elderly. The limitation of the current management is that most patients can only be followed up until the AAA diameter increases to a threshold, and surgical intervention is recommended. The development of preventive and curative drugs for AAA is urgently needed. Macrophage‐mediated immune inflammation is one of the key pathological links in the occurrence and development of AAA.

**Aims:**

This review article aims to evaluate the impact of immunometabolism on macrophage biology and its role in AAA.

**Methods:**

We analyze publications focusing on the polarization and metabolic reprogramming in macrophages as well as their potential impact on AAA, and summarize the potential interventions that are currently available to regulate these processes.

**Results:**

The phenotypic and functional changes in macrophages are accompanied by significant alterations in metabolic pathways. The interaction between macrophage polarization and metabolic pathways significantly influences the progression of AAA.

**Conclusion:**

Macrophage polarization is a manifestation of the gross dichotomy of macrophage function into pro‐inflammatory killing and tissue repair, that is, classically activated M1 macrophages and alternatively activated M2 macrophages. Macrophage functions are closely linked to metabolic changes, and the emerging field of immunometabolism is providing unique insights into the role of macrophages in AAA. It is essential to further investigate the precise metabolic changes and their functional consequences in AAA‐associated macrophages.

## Graphical abstract

This review highlights the importance of macrophage polarization and metabolic reprogramming in abdominal aortic aneurysm and summarizes the potential interventions that are currently available to regulate these processes.

## 1 INTRODUCTION

Abdominal aortic aneurysm (AAA) is a potentially fatal disease with a significant mortality rate in the event of rupture.^1^ In recent years, advances have been made in surgical and endovascular repair of AAA. However, there is no pharmacological therapy available to prevent the enlargement and rupture of AAA.^2^ More research is needed to understand the pathological mechanisms that cause AAA formation, maintain its growth, and accelerate its rupture. AAA is defined as the dilation of the adjacent vessels by more than 50% of their normal diameter or when the diameter of the abdominal aorta reaches 30 mm.^3^ AAA is characterized by local infiltration of inflammatory cells, degradation of extracellular matrix by protein hydrolases, loss and phenotypic transformation of vascular smooth muscle cells,^4^ of which immunocytes, especially macrophages, play an important role in the incidence and progression of AAA, making the emerging field of immunometabolism a new entry point to elucidate the pathogenesis of AAA.^5^

Macrophages are essential components of innate immunity, and their remarkable plasticity enables them to execute a wide range of tasks in response to challenges posed by tissue physiology or environmental changes.^6^ When the environment changes, macrophages can be polarized in different directions, thereby regulating their immunological activity.^7^ Recent studies have increasingly shown that activated macrophages exhibit significant metabolic changes that influence their immune functions. This metabolic reprogramming and its functional consequences in cancer are well known. Metabolic reprogramming holds great potential for the functional regulation of macrophages and the development of new therapeutic approaches.^8^ However, its involvement in the initiation, progression, and treatment of AAA requires further investigation. This article reviews the polarization and metabolic reprogramming in macrophages, and the potential impact on AAA will be discussed.

## 2 ORIGIN OF MACROPHAGES

Macrophages were originally defined as phagocytic cells by Élie Metchnikoff.^9^ A widely accepted definition proposes the mononuclear phagocyte system, which includes blood monocytes, tissue macrophages, and monocyte‐derived dendritic cells, and tissue macrophages are thought to be derived from circulating blood monocytes.^10^ According to this model, adult tissue macrophages were defined as terminal cells of the mononuclear phagocytic lineage derived from circulating monocytes in the bone marrow.^6^ However, recent studies have shown that not only are tissue‐resident macrophages largely embryonic‐derived, but also that macrophages locally proliferate in adult organisms to maintain the resident population. The renewal of tissue‐resident macrophages is significantly influenced by their local environment.^11^ During homeostasis, colony‐stimulating factor 1 appears to be sufficient to recruit and differentiate most tissue macrophages, most likely originating from LY6C^−^ blood monocytes. In response to inflammation, LY6C^+^ blood monocytes are recruited from the blood and differentiate into inflammatory macrophages. At this stage, macrophages exhibit enhanced phagocytic, pro‐inflammatory, and destructive properties. Depending on the inflammatory environment, these macrophages can polarize into specific phenotypes, such as M1 or M2 macrophages.^10^

## 3 ORIGIN OF ARTERIAL MACROPHAGES

Research indicates that the majority of tissue‐resident macrophages in various tissues do not require continuous input from circulating monocytes and can be maintained by self‐renewal. Arterial macrophages are derived from CX3CR1^+^ precursors during embryogenesis and from monocytes in the bone marrow after birth. These monocytes colonize the tissues shortly after birth. Arterial macrophages are maintained by CX3CR1–CX3CL1 interactions and local proliferation rather than by recruitment from circulating monocytes. Self‐renewal allows arterial macrophages to return to a functional state after severe depletion caused by various septic conditions.^12^ In an experiment with ApoE^−/−^ mice fed a high‐fat diet, macrophage replenishment depended primarily on local macrophage proliferation rather than monocyte recruitment.^13^

Although tissue macrophages are self‐renewing, stimulation of local vascular tissue can lead to the release of chemokines and inflammatory cytokines that recruit circulating monocytes into the vessel wall. Both tissue‐resident macrophages and macrophages derived from circulating monocytes infiltrating damaged vessels can undergo cell proliferation.^14^ Tissue‐resident macrophages have recently been identified as essential for maintaining arterial wall homeostasis by regulating smooth muscle cell activity and collagen production.^15^ Tissue‐resident macrophages express the lymphatic vessel endothelial hyaluronan receptor‐1 (Lyve‐1),^16^ but studies on the role of tissue‐resident macrophages in AAA formation and progression are limited.

Ulndreaj et al. demonstrated that AAA was associated with a decreased percentage of Lyve‐1^+^ macrophages and depletion of Lyve‐1^+^ macrophages worsened AAA in Ang II/β‐aminopropionitrile‐induced AAA.^16^ Al‐Rifai et al. found that JAK2V617F mutation was associated with a pro‐inflammatory phenotype of perivascular tissue‐resident macrophages, which promoted adverse aortic remodeling at early stages, and AAA formation through the recruitment of circulating monocytes at later stages.^17^ It suggests that vascular tissue‐resident Lyve‐1^+^ macrophages protect the aorta from deleterious remodeling, but could be induced to promote pathological progression of AAA.

## 4 MACROPHAGE POLARIZATION AND AAA

### 4.1 Macrophage polarization

Precise regulation of macrophage activation is crucial for controlling diseases and maintaining tissue homeostasis.^7^ Macrophage polarization is closely associated with metabolic reprogramming. Due to the high plasticity of macrophages, their phenotype changes in response to variations in the microenvironment and can be categorized into two main types: classically activated or M1 macrophages activated by lipopolysaccharide (LPS) with or without IFN‐γ, secreting interleukin (IL)−1β, IL‐12, and expressing surface markers of cluster of differentiation (CD) 80, CD86 and alternatively activated or M2 phenotype activated by IL‐4/IL‐10, secreting IL‐4, IL‐10, and expressing surface markers CD68, CD206, CD163.^18^, ^19^ Although the classification of macrophages into M1 and M2 subsets may oversimplify their diversity, it is a critical factor in examining macrophage activation and metabolic responses in inflammatory illnesses.

It has been reported that M2 macrophages are more highly expressed in the intraluminal thrombi of human AAA specimens, whereas M1 macrophages predominate in the AAA epicardium and may promote aneurysm development.^20^ In contrast, Dutertre et al. found the opposite by examining the composition and distribution of stromal and hematopoietic cells in the adventitia of human aortas, with a significant decrease in M1 and an increase in M2 in the adventitia of AAA compared to non‐AAA.^21^ This may be explained by differences in the markers that differentiate between M1 and M2. The samples obtained for AAA often represent the end stage of the disease. The macrophage phenotype shifts at different stages of disease progression due to the complex regulation of the body. However, this does not diminish the importance of macrophage polarization in AAA development. Maintaining the M1/M2 ratio within a certain range is essential for aortic tissue homeostasis and provides new ideas for AAA treatment.

### 4.2 Macrophage polarization and its crucial role in regulating AAA formation

**Figure 1:**
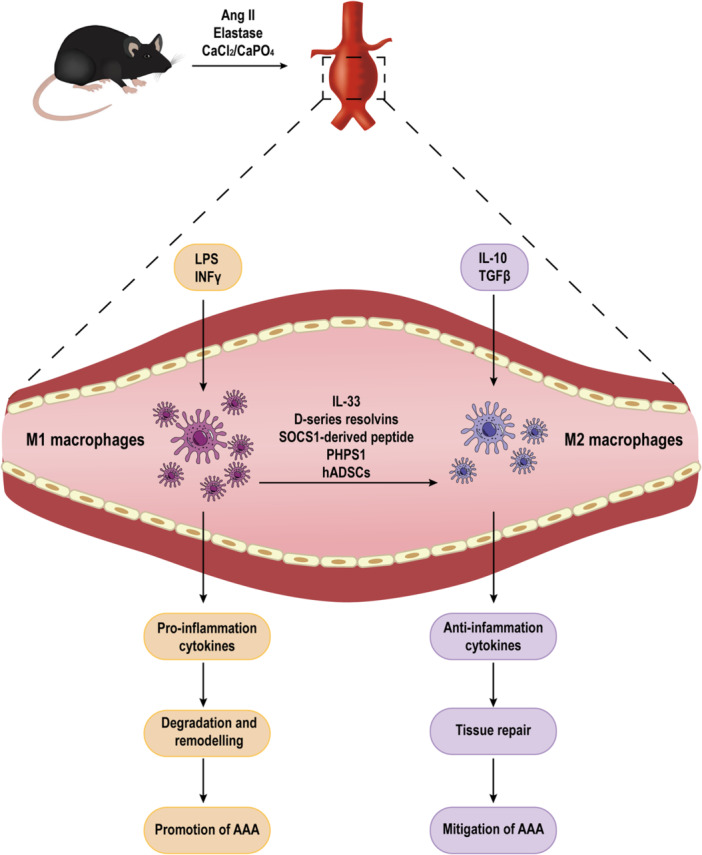
Kinetics graph of M1 and M2 macrophages accumulation in animal abdominal aortic aneurysm (AAA) models. The mouse AAA model can be established mainly by angiotensin II (Ang II), elastase and calcium chloride (CaCl^2^)/calcium phosphate (CaPO_4_), M1 macrophages in AAA can be activated by lipopolysaccharide (LPS)/interferon‐gamma (INFγ), releasing pro‐inflammatory cytokines, which lead to the destruction of the aortic wall, degradation of extracellular matrix and pathological remodeling, aggravating the progression of AAA. M2 macrophages can be activated by interleukin (IL)‐10 and transforming growth factor beta (TGFβ), releasing anti‐inflammatory cytokines, which can promote tissue repair and alleviate the progression of AAA. Currently, it has been found that IL‐33, d‐series resolvins, suppressor of cytokine signaling mimetic peptide (SOCS1)‐derived peptide, PHPS1, xenotransplantation of human adipose‐derived stem cells (hADSCs), and so on, can promote the polarization of M1–M2 and inhibit the development of AAA. PHPS1, phenylhydrazonopyrazolone sulfonate 1.

The role of macrophages in the progression of AAA is widely acknowledged, and their ability to modulate inflammation through polarization can impact the development and progression of AAA, which is closely related to pathological processes such as extracellular matrix degradation and remodeling and oxidative stress imbalance (Figure 1). In the context of AAA, immune cells infiltrate the arterial wall, where they adopt different phenotypes based on their microenvironment. M1 macrophages respond to environmental stimuli and sustain ongoing inflammation by producing proteolytic enzymes and pro‐inflammatory mediators. Conversely, M2 macrophages are associated with wound healing and inflammation abatement. Therefore, the balance between M1 and M2 macrophages in the AAA microenvironment is crucial for the disease progression (Table 1).^22^

**Table 1:** Summary of the targets reported on macrophage polarization and AAA.

Targets and intervention	Model used	Effects on macrophages	Effect on AAA	Ref.
Macrophage‐specific Sirt1 knockout mice	Ang II infusion	M1↑M2↓	Enhanced AAA formation	[23]
IL‐33	CaPO_4_‐induced; Elastase‐induced	M2↑	Reduced AAA formation	[24]
*Il‐18* ^*−/−*^ mice	Ang II + BAPN	M2↑	Reduced AAA formation	[25]
*Tnf‐α* ^*−/−*^mice	CaCl_2_‐induced	M1↓	Reduced AAA formation	[26]
TAPI‐1 (ADAM17 inhibitor)	CaPO_4_‐induced	M1↓	Inhibited the formation and development of AAA	[27]
Anti‐TGF‐β	Elastase‐induced	M2↑	Further studies are required to elucidate its role in AAA	[28]
d‐series resolvins	Elastase‐induced; Ang II infusion	M2↑	Inhibited AAA formation	[29]
PHPS1 (SHP2 inhibitor)	Ang II infusion	M2↑	Inhibited AAA formation	[30]
SOCS1‐derived peptide	Elastase‐induced	M1↓M2↑	Inhibited AAA formation	[31]
CCL7‐neutralizing antibody	Ang II infusion	M1↓	Inhibited AAA formation	[32]
S3I‐201 (small molecule STAT3 inhibitor)	Ang II infusion	M1↓M2↑	Inhibited AAA formation	[33]
TLR4 inhibitor Eritoran; *Tlr4* ^−/−^ mice	Ang II infusion	M1↓M2↑	Inhibited AAA formation	[33]
DAPT, inhibitor of Notch signaling	Ang II infusion	M1↓	Inhibited AAA formation	[34, 35]
*Notch1* ^+/−^ mice	Ang II infusion	M1↓M2↑	Inhibited AAA formation	[36]
Endogenous bioactive peptide intermedin	Ang II infusion; CaCl_2_‐induced	M1↓M2↑	Inhibited AAA formation	[37]
hADSCs	Elastase‐induced	M1↓M2↑	Inhibited AAA formation	[38]
Mice deficient in GM‐CSF (*Csf2* ^−/−^)	CaPO_4_‐induced; elastase‐induced	M1↓	Inhibited AAA formation	[39]
Adoptive transfer of Tregs	Ang II infusion	M1↓M2↑	Inhibited AAA formation	[40]
Eosinophil‐deficient Apoe^−/−^ΔdblGATA mice	Ang II infusion	M1↑	Enhanced AAA formation	[41]
miR‐24	Elastase‐induced; Ang II infusion	M1↓	Inhibited AAA formation	[42]
miR‐144‐5p	Ang II infusion	M2↑	Inhibited AAA formation	[43]
CircCdyl	Ang II infusion; CaCl2‐induced	M1↑	Enhanced AAA formation	[44]
miR‐223‐loaded nanoparticles	Ang II infusion	M1↓M2↑	Inhibited AAA formation	[45]

Abbreviations: AAA, abdominal aortic aneurysm; ADAM17, a disintegrin and metalloproteinase‐17; BAPN, β‐Aminopropionitrile; CCL7, chemokine C‐C motif ligand 7; circCdyl, circular RNA Cdyl; DAPT, (*N*‐[*N*‐(3,5‐difluorophenacetyl)‐l‐alanyl]‐*S*‐phenylglycine t‐butyl ester); EOS, eosinophils; GM‐CSF, granulocyte‐macrophage colony‐stimulating factor; hADSCs, xenotransplantation of human adipose‐derived stem cells; IL, interleukin; IMD, intermedin; M1, classically activated macrophages; M2, alternatively activated macrophages; PHPS1, phenylhydrazonopyrazolone sulfonate 1; SHP2, tyrosine phosphatase‐2; SIRT1, sirtuin type 1; SOCS1, suppressor of cytokine signaling mimetic peptide; STAT, signal transducer and activator of transcription; TAPI‐1, TNF‐α processing inhibitor‐1; TGF‐β, transforming growth factor beta; TLR4, Toll‐like receptor 4; TNF‐α, tumor necrosis factor alpha; Tregs, regulatory T cells.

Sirtuin type 1 (SIRT1), a member of the class III deacetylase family, acts as an anti‐inflammatory factor in peritoneal macrophages by reducing cyclooxygenase‐2 expression and prostaglandin E_2_ (PGE_2_) production.^46^ Small molecule activator of SIRT1 enhances cellular p65 protein deacetylation, which inhibits tumor necrosis factor (TNF)‐α‐induced transcriptional activation of nuclear factor kappa B (NF‐κB),^47^ a key transcription factor for macrophage M1 activation. In the angiotensin II (Ang II)‐induced AAA model, macrophage‐specific *Sirt1* knockout mice showed that SIRT1 deficiency promotes M1 polarization and reduces M2 polarization, thereby contributing to AAA formation.^23^

IL‐33, a member of the IL‐1 cytokine family, is secreted by infiltrating inflammatory cells at the site of inflammation. By inducing ST2 (the receptor for IL‐33) dependent Treg proliferation, IL‐33 protects against AAA development in mice by reducing macrophage infiltration and promoting polarization of lesional macrophages toward the M2 phenotype.^24^ Additionally, IL‐18, another member of the IL‐1 family, regulates osteopontin production. Its deficiency can shift macrophages from a pro‐inflammatory M1 state to an anti‐inflammatory M2 state, thereby attenuating AAA formation.^25^

IL‐1β and TNF‐α are considered typical inflammatory cytokines. Polarization of M1 macrophages (M1/M2 imbalance) is critical for AAA formation in the CaCl_2_ model.^48^ In the elastase infusion model, Johnston et al found that AAA formation was reduced by either suppression or antagonism of IL‐1β.^49^ Xiong et al. have shown that the inhibition or absence of TNF‐α can affect macrophage infiltration and prevent AAA formation.^50^ To clarify that IL‐1β inhibition is similar to TNF‐α antagonism for suppression, they were surprised to find that IL‐1β deficiency in the CaCl_2_ model does not block M1 macrophage polarization and does not inhibit AAA formation, whereas TNF‐α deficiency tends to polarize them toward M2, reducing the destructive effects of M1 macrophages. Therefore, in many inflammatory conditions, IL‐1β inhibition is less effective than TNF‐α inhibition, and this discrepancy may be due to differences between the models.^26^ ADAM17, also known as TNF‐α converting enzyme, is increased in AAA. Inhibition of ADAM17 can reduce M1 macrophages and inhibit AAA formation.^27^ Transforming growth factor‐β (TGF‐β) activity is necessary for healing of AAA.^51^ Neutralization of TGF‐β can fine‐tune macrophage phenotypes and increase the number of M2 macrophages expressing arginase‐1 (Arg‐1) in the aortic wall in elastase‐induced AAA.^28^

In a mouse model of AAA induced by Ang II, the single‐cell RNA sequencing technology showed a gradual decrease in the number of M2 macrophages as the disease progressed, indicating a polarization toward a pro‐inflammatory phenotype.^52^ Another study using a combination of single‐cell RNA sequencing, weighted gene co‐expression network analysis, and differential expression analysis, showed that M1 and M2 macrophages exert a resistance effect in small AAA (mean maximum aortic diameter ≤55 mm). This resistance effect makes them potential therapeutic targets for controlling inflammation, mitigating arterial wall destruction, and reducing AAA expansion and rupture.^53^ The transition to an M2 phenotype in terminal‐stage AAA may be a compensatory mechanism to prevent AAA expansion and rupture.^54^

Estrogen has been found to upregulate the expression of TROVE2, and promote the conversion of macrophages to the M2 phenotype, thereby providing protection against AAA.^55^ d‐series resolvins, particularly resolvin D, have been shown to decrease M1 and increase M2 markers expression without affecting the absolute number of infiltrating macrophages, which suggests a role in polarization of activated macrophages.^56^ Likewise, resolvin D can increase M2 macrophage polarization and inhibit AAA in mice in another article.^29^

There are conflicting results regarding tyrosine phosphatase‐2 (SHP2) and the regulation of macrophages. In the inflammatory environment, SHP2 inhibition can enhance inappropriate production of interferon (IFN)‐β and pro‐inflammatory cytokines in macrophages, promoting the development of immunopathological diseases.^57^ On the other hand, another study has shown that SHP2 inactivation enhances IL‐4‐mediated M2 polarization in an immunosuppressive environment.^58^ Lung epithelial SHP2 deficiency reduces IL‐8 release and lung inflammation in mice with lung disease.^59^ It is hypothesized that SHP2 may directly influence the immune status of macrophages, which ultimately determines the direction of AAA. phenylhydrazonopyrazolone sulfonate 1 (PHPS1), an SHP2 inhibitor, inhibits the expression of IFN‐γ, TNF‐α, and matrix metalloproteinases while increasing the expression of IL‐10 and Arg‐1, inducing macrophage polarization toward the M2 type, thereby attenuating AAA progression. SHP2 could be a therapeutic target for AAA, and PHPS1 might be a potential compound to halt AAA progression.^30^

Signal transducer and activator of transcription (STAT) plays a crucial role in macrophage polarization, with M1‐type polarization being closely linked to STAT1, while M2‐type macrophage polarization is affected by increased expression of STAT3 and STAT6.^60^ Four kinases, Janus kinase (JAK) 1–3 and tyrosine kinase 2 and seven transcription factors (STAT1‐4,5A, 5B, and 6), comprise the JAK/STAT family, and the suppressor of cytokine signaling (SOCS) family (SOCS1‐7 and cytokine‐inducible SH2 protein) act as negative feedback inhibitors to shut down the signaling cascade.^61^ In an elastase‐induced mouse model of AAA, researchers mimicked the SOCS1 kinase inhibitory structural domain with a synthetic cell permeable‐peptide (S1) to inhibit STAT activation, and found that the S1 peptide inhibited aortic M1 macrophages while promoting the expression of M2 macrophages‐associated markers, and downregulating the pro‐inflammatory cytokines. SOCS1 therapy contributes to AAA improvement by biasing macrophages toward an M2 phenotype for repair of damaged tissue in the study.^31^ In addition, research has revealed that chemokine C–C motif ligand 7 (CCL7) promotes AAA by enhancing macrophage migration and transition to an M1 phenotype via binding to C–C chemokine receptor type 1 and activation of the JAK2/STAT1 pathway.^32^ The small molecule STAT3 inhibitor S3I‐201 reduced Ang II‐induced AAA formation and severity by decreasing the M1/M2 macrophage ratio.^33^

Notch receptors are highly conserved signaling pathways crucial for development and involved in malignant transformation. In mammals, there are four Notch receptors (Notch1–4).^62^ Studies have indicated the important regulatory role of Notch signaling in macrophage polarization. Notch signaling inhibitor, (*N*‐(*N*‐[3,5‐difluorophenacetyl]‐l‐alanyl)‐S‐phenylglycine t‐butyl ester) (DAPT), weakens the active AAA phenotype by promoting M1–M2 transition.^34^ Notch1 deficiency promotes M2 differentiation in bone marrow‐derived macrophages by upregulating TGFβ2 and responding to IL‐4.^35^ Notch1 inhibition reduces M1 polarization and promotes M2 fate, leading to defects in macrophage migration and proliferation. Lack of Notch1 preserves the anti‐inflammatory environment and normal aortic wall structure, reducing abdominal aortic expansion.^36^ The vascular endogenous bioactive peptide intermedin (IMD) inhibits the Notch1 signaling pathway, preventing NLRP3 (NOD‐, LRR‐, and pyrin domain‐containing protein 3) activation and M1 polarization, and promoting M2 macrophage polarization, thereby reducing local aortic inflammation and preventing AAA formation.^37^

Xenotransplantation of human adipose‐derived stem cells (hADSC) induces M2 macrophage and Treg phenotypes, thereby reducing aortic inflammation and weakening aortic expansion in a murine AAA model.^38^ CD4^+^ T cells, including regulatory T cells (Tregs) and conventional T cells (Tconvs), play a role in AAA progression. Tconvs recruited to the aorta via the C–X–C chemokine receptor type 6/Chemokine (C–X–C motif) ligand 16 axis express high levels of granulocyte‐macrophage colony‐stimulating factor (GM‐CSF). GM‐CSF increases inflammatory monocyte infiltration into the aorta, inducing macrophage polarization toward M1 phenotype, which exacerbates AAA.^39^ Tregs induce macrophage transition from M1 to M2 phenotype and prevent AAA by inhibiting the conversion of arachidonic acid to PGs through the key enzyme COX‐2.^40^ Eosinophils (EOS) accumulate in human and mouse abdominal aortas, polarize macrophages to the M2 phenotype, and exert a protective effect in AAA by releasing IL‐4 and cationic proteins such as EOS‐associated‐ribonuclease‐1.^41^ Understanding these complex interactions and signaling pathways provides valuable insights for potential therapeutic interventions aimed at modulating macrophage polarization and controlling AAA development.

Noncoding RNAs, including microRNAs (miRNAs), long noncoding RNAs (lncRNAs), and circular RNAs (circRNAs), have been implicated in the regulation of AAA and macrophage polarization. MiRNAs, small noncoding RNAs consisting of 21–23 nucleotides, bind to the 3′ untranslated region of specific messenger RNA (mRNA) targets, inducing their degradation or translation suppression.^63^ Accumulating evidence supports the critical role of miRNAs in modulating the phenotypic polarization of macrophages.^64^ Studies have shown that manipulating miRNA expression, such as miR‐712, miR‐29b, and miR‐181b, can influence the progression of AAA in animal models.^65^, ^66^, ^67^ MiRNAs also affect macrophage polarization in the context of AAA. For example, miR‐24 primarily influences M1 macrophages without affecting M2 subtypes.^42^ MiR‐144‐5p exhibits protective effects in AAA formation by targeting Toll‐like receptor 2 and ox‐LDL receptor 1, which are associated with the M2 phenotype, and its anti‐AAA effect may be related to its inhibition of M1 macrophage polarization.^43^ LncRNAs, acting as signals, decoys, guides, and scaffolds, control gene expression.^68^ Overexpression of lncRNA H19 promotes AAA formation by enhancing vascular pro‐inflammatory IL‐6 and macrophage infiltration.^69^ CircRNAs, besides being major regulators of gene expression, hold potential as novel biomarkers due to their resistance to exonucleases and long half‐lives.^70^ CircRNA studies on microarray platforms have indicated their value in macrophage differentiation and polarization processes.^71^ CircCdyl, identified as a circRNA rich in M1 macrophages, significantly induces AAA formation by promoting vascular inflammation and M2 polarization through inhibiting interferon regulatory factor 4 nuclear entry.^44^

Macrophage polarization plays a crucial role in AAA and has spurred the development of nanomaterial‐based therapies. By exploiting the inherent ability of monocytes and macrophages to engulf foreign particles, nanoparticle formulations have emerged as a targeted and specific treatment option. For instance, miR‐223‐loaded nanoparticles promote the polarization of bone marrow‐derived macrophages toward the M2 phenotype, reducing the proportion of M1 macrophages and the expression of NLRP3 inflammasomes, thereby lowering the incidence of AAA.^45^

### 4.3 Lifestyle and macrophage polarization in AAA

Diet patterns on the whole and some nutrients are shown to influence macrophage polarization, including dietary fiber, polyunsaturated/monounsaturated fatty acids vitamins, and polyphenols.^72^ Grape‐seed polyphenols administered intragastrically could inhibit AAA in mice via regulation of macrophage polarization.^73^ Interesting, a prospective study suggested that a healthy diet did not reduce the risk of AAA, meantime exercising for >40 min per day reduced the risk of AAA by 41% compared to those who never exercised.^74^ The possible explanation is that a single session of exercise could potentiate the anti‐inflammatory phenotype and promote M2 polarization of macrophages.^75^ In studies of macrophage polarization, smoking has been found to be associated with altered macrophage function. In animal models of AAA, exposure to cigarette smoke accelerates the formation and severity of AAA,^76^, ^77^ and nicotine e‐cigarettes significantly increase AAA formation and enhance macrophage expression of pro‐inflammatory givers and reactive oxygen species (ROS) production,^78^ and more research is needed in this area to elucidate the specific link between cigarette smoking and macrophage polarization, and how this link may affect the pathogenesis of AAA. pathogenesis.

## 5 MACROPHAGE METABOLIC REPROGRAMMING AND ITS ROLE IN AAA

**Figure 2:**
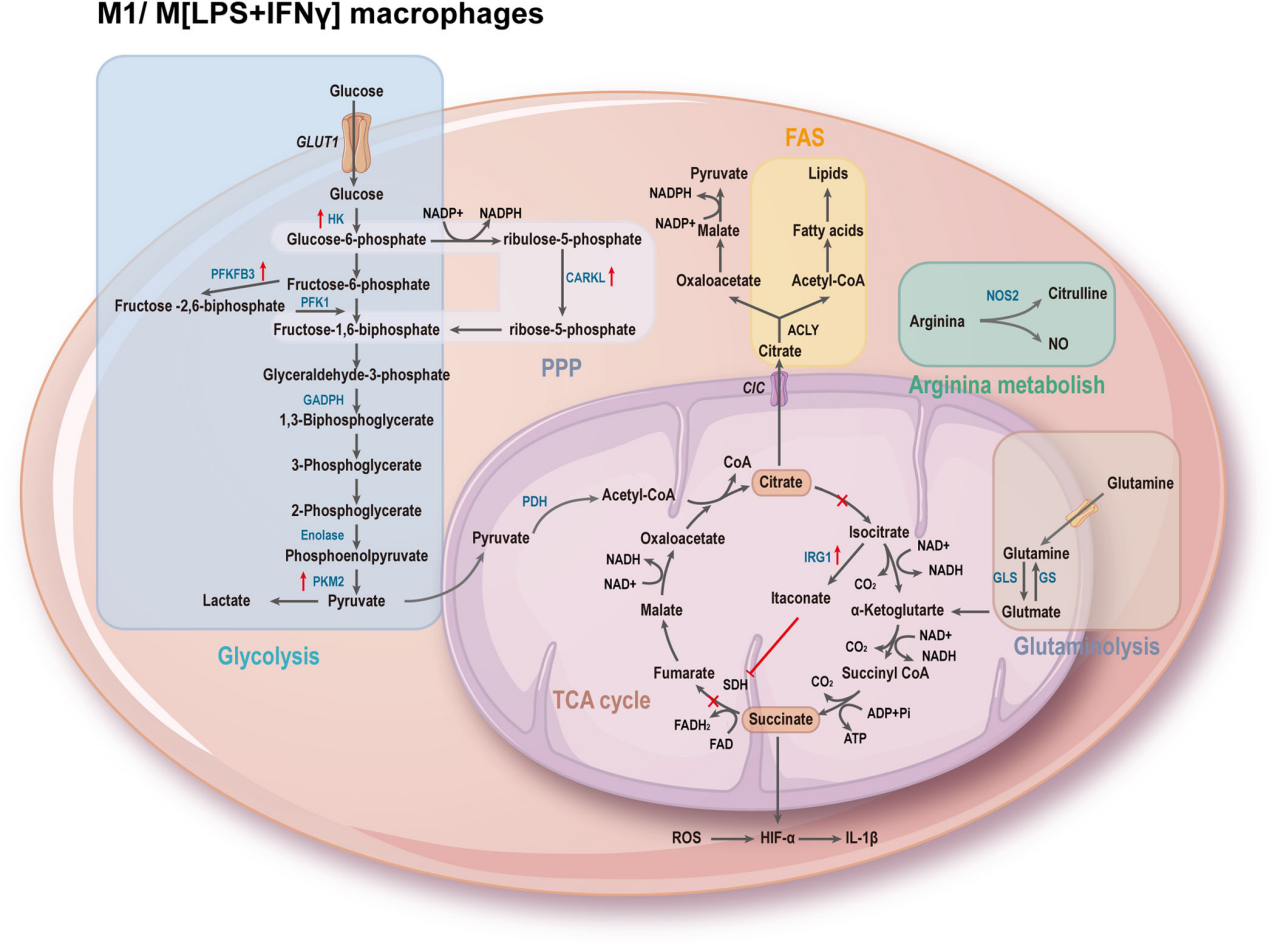
Metabolic reprogramming of M1/M [lipopolysaccharide (LPS) +  interferon‐gamma(IFN‐γ)] macrophages. In M1‐like macrophages are distinguished by enhanced aerobic glycolysis, pentose phosphate pathway (PPP), and fatty acid synthase (FAS); disruption of the tricarboxylic (TCA) cycle (after citrate and succinate respectively); and catabolism of arginine to citrulline and nitric oxide (NO). ACLY, ATP citrate lyase; ADP, adenosine diphosphate; ATP, adenosine triphosphate; CARKL, carbohydrate kinase‐like protein; CIC, citrate/isocitrate carrier; CO_2_, carbon dioxide; FAD, flavin adenine dinucleotide; FAS, fatty acid synthase; GAPDH, glyceraldehyde‐3‐phosphate dehydrogenase; GLS, glutaminase; Glut1, glucose transporter 1; GS, glutamine synthetase; HIF‐1α, hypoxia‐inducible factor 1α; HK, hexokinase; IL‐1β, interleukin‐1 beta; Irg1, immune‐responsive gene 1; NADH, nicotinamide adenine dinucleotide; NADPH, nicotinamide adenine dinucleotide phosphate; NO, nitric oxide; NOS, nitric oxide synthase; PDH, pyruvate dehydrogenase; PFK1, phosphofructokinase‐1; PFKFB3, 6‐phosphofructo‐2‐kinase/fructose‐2,6‐biphosphatase 3; PKM2, pyruvate kinase; ROS, reactive oxygen species; SDH, succinate dehydrogenase.

**Figure 3:**
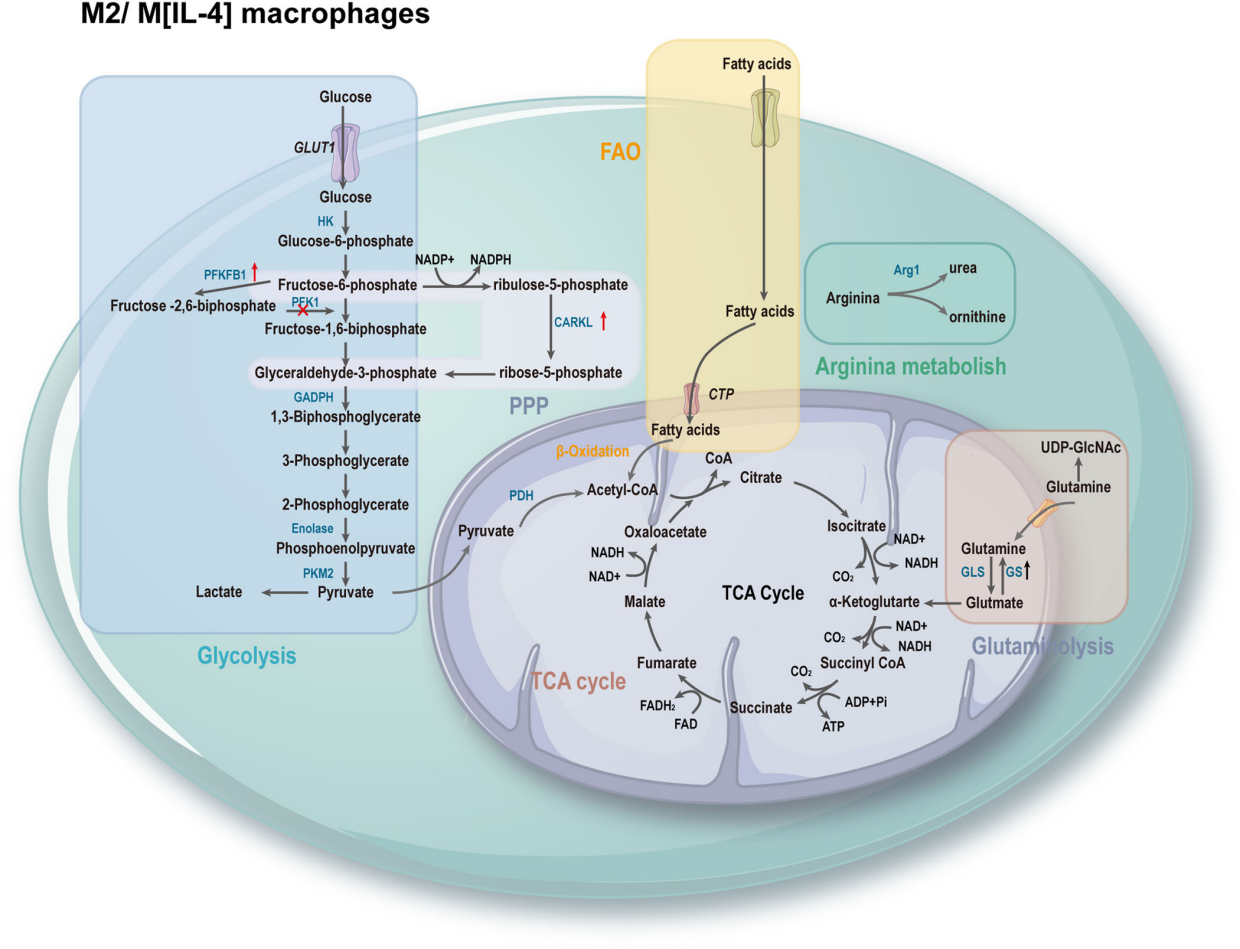
Metabolic reprogramming of M2/M [interleukin‐4 (IL‐4)] macrophages. In M2‐like macrophages exhibit an intact tricarboxylic acid (TCA) cycle, high oxidative phosphorylation (OXPHOS) rates, enhanced fatty acid oxidation (FAO), and hydrolysis of arginine to ornithine and urea. ADP, adenosine diphosphate; Arg1, arginase‐1; ATP, adenosine triphosphate; CARKL, carbohydrate kinase‐like protein; CO_2_, carbon dioxide; CTP, cytidine triphosphate; FAD, flavin adenine dinucleotide; GAPDH, glyceraldehyde‐3‐phosphate dehydrogenase; GLS, glutaminase; Glut1, glucose transporter 1; GS, glutamine synthetase; HK, hexokinase; NADH, nicotinamide adenine dinucleotide; NADPH, nicotinamide adenine dinucleotide phosphate; PDH, pyruvate dehydrogenase; PFK1, phosphofructokinase‐1; PFKFB3, 6‐phosphofructo‐2‐kinase/fructose‐2,6‐biphosphatase 3; PKM2, pyruvate kinase; PPP, pentose phosphate pathway; UDP‐GlcNAc, uridine diphosphate *N*‐acetylglucosamine

The process of macrophage polarization is accompanied by a reprogramming of energy utilization, allowing macrophages to exhibit different polarization functional characteristics.^7^ M1 macrophages exhibit enhanced glycolytic metabolism, pentose phosphate pathway (PPP), fatty acid synthesis (FAS), tricarboxylic acid (TCA) cycle, and mitochondrial oxidative phosphorylation (OXPHOS). Interruption of the TCA cycle leads to the accumulation of metabolites, many of which have signaling functions, such as citrate, succinate, fumarate, and α‐ketoglutarate (αKG). Moreover, upregulation of inducible nitric oxide synthase (iNOS) results in the degradation of arginine to citrulline and nitric oxide (NO), which plays a crucial role in intracellular pathogen killing (Figure 2). M2 macrophages rely on fatty acid oxidation (FAO), intact TCA cycle, high OXPHOS rate, increased expression of Arg‐1, leading to the production of urea, polyamines, and ornithine, promoting wound healing (Figure 3).^79^ Metabolic reprogramming reflects the functional adaptation of cell to critical environmental changes. Understanding the intricate interplay between these metabolic pathways and macrophage polarization in AAA can provide valuable insights for targeted therapeutic interventions.

### 5.1 Glycolysis

Glycolysis is a metabolic pathway controlled by several glycolytic enzymes that convert glucose to pyruvate. It was once thought to occur only under anaerobic conditions until Warburg observed that even in the absence of oxygen limitation, cells preferentially utilize glycolysis to produce adenosine triphosphate (ATP), a process known as aerobic glycolysis or the Warburg effect.^80^ Interestingly, after stimulating macrophages with LPS, a similar Warburg effect occurs with a switch from OXPHOS to aerobic glycolysis, similar to the metabolic behavior observed in tumor cells.^81^ Despite being an inefficient pathway for ATP production (one unit of glucose yields two molecules of ATP), the impact of glycolysis on macrophage metabolism extends beyond the provision of limited energy. It also involves the reduction of nicotinamide adenine dinucleotide (NAD)^+^ to NADH, with NADH serving as a cofactor for enzymes crucial in biosynthesis, including nucleotides (conversion of glucose‐6‐phosphate to ribose‐5‐phosphate), amino acids (3‐phosphoglycerate conversion to serine in the serine biosynthesis pathway), and fatty acids (conversion of 3‐phosphoglycerate to serine in the serine biosynthetic pathway), playing a key role in supporting synthetic growth.^82^, ^83^

Compared to M1 macrophages, M2 macrophages tend to favor OXPHOS, which is a slower process but yields more ATP (approximately 30 ATP per glucose molecule). When OXPHOS is intact, glycolysis is not essential for M2 activation, but it becomes necessary when OXPHOS is compromised.^84^ Key enzymes of glycolysis, including hexokinase (HK), glyceraldehyde‐3‐phosphate dehydrogenase (GAPDH), and pyruvate kinase M2 (PKM2), play essential roles in macrophage function. These enzymes are not only components of glycolysis, but also involved in complex additional functions, earning them the title of moonlighting proteins. Due to their diverse functionalities, they have the potential to be relevant targets for disease treatment.^85^

#### 5.1.1 HK

HK regulates the first step of glucose metabolism, catalyzing the conversion of glucose to glucose‐6‐phosphate.^86^ Numerous studies have demonstrated that 2‐deoxyglucose (2‐DG) competitively and allosterically inhibits HK, thereby reducing lactate production and ATP synthesis in glycolysis. 2‐DG can also lower succinate levels to inhibit LPS‐induced IL‐1β production, reducing glycolytic reactions, and can be used to treat inflammatory diseases.^87^ Furthermore, HK has been shown to effectively act as a pattern recognition receptor that activates inflammatory responses by disrupting glycolytic pathways and mitochondrial function.^88^

#### 5.1.2 GAPDH

GAPDH catalyzes the conversion of glyceraldehyde‐3‐phosphate to 1,3‐bisphosphoglycerate by reducing NAD to NADH. GAPDH can bind to NAD or NADH, and it also has multiple interactions with DNA or RNA. Apart from its role as a glycolytic enzyme, GAPDH participates in various cellular processes, including acting as a transcription regulatory factor,^89^ membrane fusion, cellular cytoskeleton dynamics, interacting with transferrin,^90^ translocating to the nucleus during apoptosis,^91^ DNA replication, and repair, maintaining DNA integrity through telomere binding, nuclear transfer RNA export, mRNA stability, controlling gene transcription and translation.^92^, ^93^

#### 5.1.3 Enolase

Enolase catalyzes the conversion of 2‐phosphoglycerate to phosphoenolpyruvate. It anchors to the cell membrane and acts as a receptor that activates plasminogen, stimulating cell migration and invasion capabilities.^94^ Enolase also has multiple binding capabilities with DNA and mRNA.^95^ Under inflammatory conditions, it undergoes posttranslational modifications such as guanidinylation and OXPHOS.^96^

#### 5.1.4 PKM2

PKM2 is the rate‐limiting enzyme in glycolysis, catalyzing the conversion of phosphoenolpyruvate and ADP to pyruvate and ATP. The tetrameric form of PKM2 functions primarily as pyruvate kinase and regulates glycolysis. The less active dimeric form of PKM2 allows the accumulation of glycolytic intermediates.^97^ PKM2 translocates to the nucleus and directly interacts with hypoxia‐inducible factor‐1α (HIF‐1α) to regulate the expression of glycolytic enzymes.^98^ Activation of PKM2 tetramers prevents nuclear translocation, inhibits HIF‐1α interaction, reverses the LPS‐induced shift toward glycolysis and IL‐1β production, and reprogrammes macrophages to an M2‐like phenotype.^99^ During periods of high demand, such as cell division, PKM2 activity is downregulated by subunit dissociation to block the flow of glycolysis toward pyruvate production. This leads to the accumulation of glycolytic intermediates that serve as precursors for nucleic acid, lipid, and amino acid synthesis. However, the dissociation of PKM2 tetramers into dimers consumes cellular ATP and activates adenosine monophosphate‐activated protein kinase (AMPK). Activated AMPK can activate p53, which, in turn, activates TP53‐induced glycolysis and apoptosis regulator (TIGAR). The TIGAR protein blocks phosphofructokinase (PFK), potentially conserving ATP consumption in this step and directing more glycolytic intermediates toward synthetic pathways involving PKM2.^100^ Regulation of PKM2 catalytic activity also modulates the synthesis of DNA and lipids necessary for cell proliferation and the nicotinamide adenine dinucleotide phosphate (NADPH) required for redox homeostasis. In addition to its role as a pyruvate kinase, PKM2 also functions as a protein kinase and a transcriptional coactivator. These biochemical activities are controlled by allosteric regulators and posttranslational modifications of PKM2, including acetylation, OXPHOS, succinylation, and sumoylation.^101^

#### 5.1.5 HIF‐1α

Increased glycolysis associated with inflammatory macrophages is highly dependent on HIF‐1α, which plays a critical role in inducing key enzymes of glycolysis.^102^ HIF is a heterodimeric transcription factor composed of α and β subunits, with a short half‐life and easy degradation under normoxic conditions. However, hypoxia inhibits prolyl hydroxylase (PHD), leading to the accumulation of HIF‐1α. LPS in combination with IFN‐γ induces metabolic reprogramming of M1 macrophages into glycolysis through HIF‐1α. In AAA, the expression of HIF‐1α and its target genes significantly increases,^103^ including in human and Ang II‐induced mouse AAA models and porcine pancreatic elastase‐induced mouse AAA models.^104^ Whether HIF‐1α affects the metabolic reprogramming of macrophages in AAA remains to be further explored.

Studies have shown that positron emission tomography/computed tomography using the glucose analog 18F‐fluorodeoxyglucose significantly increased the maximum standardized uptake value at the site of AAA, suggesting increased glycolytic activity in AAA. Furthermore, the use of 2‐DG restricted glycolysis and subsequently limited the development of AAA in a mouse model.^105^ The final rate‐limiting enzyme in glycolysis, PKM2, activated by extracellular vesicles from T lymphocytes, can be absorbed by receptor macrophages, promoting iron accumulation and lipid peroxidation in macrophages, thereby increasing their migration and promoting the progression of mouse AAA.^106^

### 5.2 PPP

PPP branches off from glycolysis and serves as a critical pathway for ribonucleotide synthesis and a major source of NADPH. The PPP consists of two phases: the oxidative phase, where glucose‐6‐phosphate undergoes dehydrogenation, hydration, and oxidative decarboxylation to produce ribulose‐5‐phosphate (Ru5P) while reducing NADP^+^ to NADPH. NADPH has multiple roles, including supporting the synthesis of NADPH oxidase to generate ROS, detoxifying ROS as a cofactor for glutathione reductase, and promoting lipid synthesis. In the non‐oxidative phase, Ru5P is converted back to glucose‐6‐phosphate, a precursor for nucleotide and amino acid synthesis.^107^ Additionally, reversible reactions can recycle Ru5P back into glycolytic intermediates such as fructose‐6‐phosphate and glyceraldehyde‐3‐phosphate or convert it into ribose‐5‐phosphate.^108^ In particular, carbohydrate kinase‐like protein (CARKL) catalyzes the production of sedoheptulose‐7‐phosphate, an intermediate in the nonoxidative phase of PPP. Studies suggest that downregulation of CARKL is crucial for M1 polarization, which appears to be essential for maintaining carbon flux in glycolysis and PPP.^109^ Conversely, M2‐like activation in macrophages leads to upregulation of CARKL.^110^

### 5.3 TCA

The TCA cycle begins with the reaction of acetyl‐CoA derived from glucose by glycolysis, or from fatty acids by β‐oxidation, or from glutamate‐derived αKG. Acetyl‐CoA, together with oxaloacetate (OAA) formed in the citrate synthase‐catalyzed reaction, forms citrate. Citrate is isomerized to isocitrate by aconitase.^111^ Isocitrate is then converted to αKG by isocitrate dehydrogenase (IDH), producing carbon dioxide and NADH in the process. αKG is further converted to succinyl‐CoA by αKG dehydrogenase, producing NADH. Succinyl‐CoA is converted to succinate by succinyl‐CoA synthetase, which simultaneously converts guanosine diphosphate to guanosine triphosphate. Succinate is oxidized to fumarate by succinate dehydrogenase (SDH), converting flavin adenine dinucleotide (FAD) to FADH2. Fumarate is then hydrated to form malate, and malate is dehydrogenated to form OAA, producing NADH. OAA is ready to combine with another acetyl‐CoA to restart the cycle.^112^ The differences in the TCA cycle observed between different macrophage subtypes are remarkable. In M2 macrophages, the TCA cycle remains intact, coupled with OXPHOS to produce ATP. However, in M1 macrophages, the TCA cycle is disrupted at two points—after citrate and after succinate, leading to the accumulation of citrate, succinate, and fumarate.^113^

#### 5.3.1 Citric acid

A recent discovery by integrated high‐throughput transcriptomics and metabolomics analysis revealed that the first metabolic perturbation in the TCA cycle in M1 macrophages may be due to the downregulation of IDH at the transcriptional level.^114^ Inhibited IDH leads to the accumulation of citrate, which is transported from mitochondria to the cytoplasm via the citrate carrier encoded by the SLC25A1 gene (citrate/isocitrate carrier). Citrate accumulation plays multiple regulatory roles. It can directly inhibit PFK 1 and 2, and indirectly inhibit pyruvate kinase, thus inhabiting glycolysis. It also stimulates lipogenesis via acetyl‐CoA carboxylase (ACC) and activates fructose‐1,6‐bisphosphatase to stimulate gluconeogenesis. Citrate is further broken down to OAA and acetyl‐CoA by ATP citrate lyase (ACLY). OAA is reduced to malate, which is then converted to pyruvate to produce NADPH.^115^ Inhibition of ACLY activity or gene silencing leads to reduced levels of inflammatory mediators such as NO, ROS, and PGE_2_.^116^ Conversely, upregulation of ACLY promotes acetyl‐CoA incorporation into histones, which increases histone acetylation and induction of genes associated with the inflammatory response.^117^

#### 5.3.2 Itaconic acid

In the context of IDH downregulation, cis‐aconitate (derived from citrate) is converted to itaconic acid by the enzyme encoded by immune‐responsive gene 1 (Irg1).^118^ Itaconic acid inhibits isocitrate lyase, which diverts citric acid cycle intermediates and displayed antibacterial properties.^119^ The inhibition of ROS, TNF‐α, IL‐6, and IFN‐β production in LPS‐tolerant macrophages further demonstrates the anti‐inflammatory role of Irg1 and itaconic acid.^120^ Itaconic acid competitively inhibits SDH, reducing succinate levels and thereby regulating macrophage metabolism and effector functions.^121^ Itaconic acid is one of the most highly induced metabolites in M1 macrophages.^121^ Current research in AAA has shown that the macrophage anti‐inflammatory pattern recognition receptor, scavenger receptor A1, can activate STAT3 phosphorylation, which further promotes Irg1 transcription, suppresses metabolic reprogramming, and inhibits inflammation, thereby exerting a protective effect against aortic aneurysm formation.^122^ Itaconic acid/Irg1 prevents AAA formation by activating nuclear factor erythroid 2‐related factor 2 (Nrf2), which inhibits the expression of downstream inflammatory genes. Exogenous administration of itaconic acid significantly reduces vascular inflammation and the rate of AAA.^123^

#### 5.3.3 Succinic acid

The second metabolic checkpoint in the TCA cycle of M1 macrophages occurs at SDH,^114^ resulting in the accumulation of succinate. Succinate accumulation activates HIF‐1α during inflammation, leading to increased IL‐1β production and a pro‐inflammatory phenotype.^87^ Succinate also induces the diversion of argininosuccinic, which ultimately replenishes citrate.^124^

### 5.4 Mitochondria function

Mitochondria are a central metabolic organelle that regulates OXPHOS and ATP production by coordinating the TCA cycle and electron transport chain (ETC).^125^ As a major site for ROS generation, the ETC consists of respiratory complexes I–IV and electron carriers. The process of oxidizing NADH and FADH2 to NAD^+^ and FAD^+^ in the ETC complexes is known as OXPHOS.^126^ Complexes I and III regulate ROS production. ROS generated by complex I can promote the expression of the pro‐inflammatory factor IL‐1β and inhibit the expression of IL‐10.^124^ Additionally, ROS produced by complex III can stabilize HIF‐1α by inhibiting PHD.^127^ Complex II can drive the reverse electron transport chain to increase ROS production and stabilize HIF‐1α. The SDH subunit of complex II also increases ROS production through competitive inhibition of SDH by clathrin from the disrupted TCA cycle in M1.^128^ ROS can induce macrophage phenotypes through different signaling pathways. ROS activates STAT‐1 and NF‐κB to promote M1 polarization.^129^ M1 macrophages inhibit OXPHOS, which inhibits their repolarization to M2 phenotype.^130^ By inhibiting iNOS, the mitochondrial function can be restored to promote M2 polarization.^131^

The pathological progression of AAA was usually accompanied by mitochondrial dysfunction.^132^ Mitochondrial ROS is the major source of the ROS‐activating NLRP3 inflammasome in macrophages.^133^ Scavenging mitochondrial ROS with the mitochondria‐targeted antioxidant Mito‐Tempo abolished the development of AAA.^134^ The mitochondria‐targeted tetrapeptide, Szeto‐Schiller 31, ameliorated mitochondrial dysfunction and limited plasmatic and vascular ROS levels, preventing experimental AAA.^135^ These findings suggest that mitochondrial ROS scavenging is insufficient during AAA development.

### 5.5 Fatty acid metabolism

The pro‐inflammatory effect of LPS‐activated macrophages is closely related to FAS.^136^ Citrate in the cytoplasm, released under the action of ACLY, is converted to acetyl‐CoA by ACC1, which is then transformed into palmitoyl‐CoA under the action of fatty acid synthase, extending into complex fatty acids.^137^ Sterol regulatory element‐binding protein‐1a not only activates the genes necessary for LPS‐stimulated macrophage activation of lipid generation but also activates the gene expression of Nlrp1a (NLR family, pyrin domain containing 1A), which produces IL‐1β and triggers the pro‐inflammatory response.^138^ Compared to M1 macrophages, M2 macrophages are more dependent on FAO. Free fatty acids are converted to acyl‐CoA by acyl synthetase and then transported into the mitochondrial matrix through carnitine palmitoyltransferas I (CPT1) bound to carnitine. Subsequently, long‐chain acylcarnitines are converted back to acyl‐CoA by carnitine CPT2 catalysis, generating acetyl‐CoA, NADH, and FADH_2_ through a series of repeated cycles.

Macrophages activated by IL‐4 or IL‐13 are dependent on FAO, a process regulated by the metabolic regulators peroxisome proliferator‐activated receptor (PPAR)γ, STAT6, and the PPARγ Coactivator 1β.^139^ In addition, CD36 takes up triglyceride substrates and subsequently undergoes lipolysis by lysosomal acid lipase, promoting OXPHOS and playing an important role in M2 activation and lifespan changes.^140^ It is worth noting that macrophages lacking CPT2 have impaired fatty acid β‐oxidation but do not affect their polarization into the M2 state, suggesting that CPT1 may have functions other than FAO in M2 macrophages.^141^ Activation of FAO through CPT1A leads to NADPH oxidase 4‐dependent production of cellular superoxide, promoting NLRP3 inflammasome activation.^142^ Hence, FAO not only has anti‐inflammatory effects but also supports inflammasome activation.^19^ Studies have suggested that eicosapentaenoic acid, a 20‐carbon omega‐3 polyunsaturated fatty acid, may inhibit the development of AAA by enhancing the anti‐inflammatory effects of M2.^143^ The specific mechanisms involved in this process warrant further investigation.

### 5.6 Amino acid metabolism

Amino acids, as the building blocks of proteins, play a crucial role in the maturation, differentiation, and function of macrophages, relying heavily on the transport and metabolism of amino acids. In a study of metabolites in AAA patients and healthy individuals by ultraperformance liquid chromatography‐tandem mass spectrometry, abnormalities in glutamate and arginine metabolism were detected,^144^ and Ang II‐induced AAA mice by metabolomics analysis found reduced glutamine and glycine concentrations and increased iNOS concentrations.^145^

#### 5.6.1 Glutamine metabolism

Glutamine is essential for the polarization and maintenance of M2 macrophage phenotype. Glutamine participates in supporting TCA cycle (one‐third of carbon in TCA metabolites of M2 macrophages comes from glutamine) and provides structural features for the synthesis of uridine diphosphate *N*‐acetylglucosamine (UDP‐GlcNAc) (more than half of the nitrogen in UDP‐GlcNAc produced by M2 macrophages comes from glutamine), which plays a crucial and specific role in M2 polarization.^114^ Glutamine synthetase (GS) can increase glutamine production and is more highly expressed in starved M2 macrophages. Inhibition of its activity leads to activation of HIF‐1α, causing succinate accumulation and activation of the M1 phenotype.^146^ Glutamine catabolism produces the anti‐inflammatory metabolite αKG, which enhances M2 activation and controls the metabolic reprogramming of M2 macrophages through the Jumonji domain‐containing protein 3‐dependent regulation. Glutamine metabolism inhibits the NF‐κB pathway through an αKG‐PHD‐dependent mechanism, thereby limiting M1 activation.^147^ PPARγ provides the molecular and metabolic basis for glutamine metabolism in IL‐4‐stimulated M2 macrophages, and IL‐4 induces PPARγ to control the feed‐forward loop of M2.^148^

#### 5.6.2 Arginine metabolism

Arginine is one of the most versatile amino acids in cells, serving not only as a precursor for protein synthesis but also for the synthesis of NO, polyamines, urea, proline, glutamate, creatine, and agmatine.^149^ M1 macrophages express iNOS, which metabolizes arginine to NO and citrulline. NO not only enhances macrophage antibacterial activity, but also prevents M1 repolarization to M2,^131^ and citrulline can be recycled for efficient NO synthesis through the citrulline‐NO cycle. The hallmark of M2 macrophages is Arg‐1, which hydrolyzes arginine to ornithine and urea. The arginase pathway limits the availability of arginine for NO synthesis, and ornithine can be further converted to polyamines and proline by ornithine decarboxylase (ODC), which is critical for cell proliferation and tissue repair.^150^ ODC restricts M1 activation and macrophage antibacterial activity through histone modification and altered chromatin formation.^151^ Arg‐1 competes with iNOS for arginine to block NO production.^152^ Therefore, these enzymes and their related metabolites, that is, iNOS, Arg‐1, play an important role in macrophage polarization and function.

#### 5.6.3 Metabolism of other amino acids

Studies have shown that serine and glycine and the related metabolic pathways are associated with inflammatory responses,^153^ with the one‐carbon unit being derived primarily from serine, either through direct uptake from the microenvironment or through the de novo synthesis of serine. In de novo synthesis, the glycolytic intermediate 3‐phosphoglycerate generates serine and glycine through a series of enzymatic reactions.^154^ Glutathione is synthesized from cysteine, glutamate, and glycine and acts as an antioxidant against ROS damage, and Rodriguez et al. elucidated that serine is required for LPS‐induced expression of IL‐1β mRNA by macrophages, where glutathione synthesis is dependent on serine‐derived glycine.^155^ Nrf2 is synthesized from serine through a series of enzymatic reactions with pro‐inflammatory cytokine genes, suppressing LPS‐induced expression of pro‐inflammatory cytokine genes in macrophages.^156^ Metabolic reprogramming of the TCA cycle is not only derived from glutamine, but also from the branched‐chain amino acids.^157^ l‐type amino acid transporter protein 1‐mediated leucine efflux activates the metabolic reprogramming of macrophage glycolysis and the production of pro‐inflammatory factors.^158^

## 6 POTENTIAL DRUGS AND MECHANISMS

By modulating different pathways in macrophages, small molecules such as dimethyl malonate, TEPP‐46 (PKM2 activator), rapamycin, 2‐deoxy‐d‐glucose, dimethyl fumarate and hyaluronic acid all promote M2 macrophage development while inhibiting M1 macrophage differentiation,^159^ which is likely responsible for the potent results when used in models of inflammatory disease, including AAA. Finally, it may be possible to target changes in macrophage metabolism for treatment. Approaches may include inhibition of iNOS, PKM2, or SDH, which would block inflammatory macrophage activation while supporting anti‐inflammatory pathways, including induction of IL‐10. disruption of PKM2 blocks activation of NLRP3 in inflammatory cytokine production by monocytes and macrophages in other inflammatory disease.^19^ A challenge is that the regulation of pathways in humans may have potential off‐target effects on other cells and tissues. Macrophage‐specific targeting of metabolic pathways may be necessary for therapeutic success. By exploiting the intrinsic ability of monocytes and macrophages to take up foreign particles, nanoparticle formulations could be considered for future therapeutic use.^160^

## 7 PERSPECTIVE AND FUTURE RESEARCH

Most current studies on macrophage metabolism and polarization still dichotomize macrophages into M1 and M2 subtypes. however, there is increasing evidence that macrophage subpopulations are much more than these two.^54^ The metabolic characterization of the activated state of non‐M1/M2 macrophages remains largely unexplored. It is necessary to analyze macrophages in vivo at the single‐cell level to characterize and elucidate these specific cellular subpopulations. In addition, there are fewer studies addressing the role of tissue‐resident macrophages in the progression of AAA, and how treatment on specific metabolic pathways would affect the immune function of this particular group of cells needs to be thoroughly investigated. Typically, once macrophages are induced to differentiate to the M1 phenotype, it is difficult to reverse them to the M2 phenotype. How we can therapeutically convert M1 macrophages into M2 macrophages in AAA patients is a major challenge. The findings described above are largely derived from studies of mouse macrophages, and it is needed to clarify how these results can be applied to the humans. For example, NO is an important determinant of the metabolic phenotype of mouse macrophages. In contrast, human macrophages produce little or no NO in vitro, while NO could control the phenotype of iNOS‐expressing human macrophages.^19^

## 8 CONCLUSION

The interaction between macrophage polarization and metabolic pathways significantly influences the progression of AAA. The phenotypic and functional changes in macrophages are accompanied by significant alterations in metabolic pathways. Specifically, M1 macrophages depend primarily on glycolysis, with two disruptions in the TCA cycle leading to the accumulation of succinate, which leads to the stabilization of HIF‐1α when excessive storage and, in turn, activates the transcription of glycolytic genes to maintain glycolytic metabolism in M1 macrophages. In contrast, M2 cells are more rely on OXPHOS with intact TCA cycle and ETC complex. In addition, both M1 and M2 macrophages have specific pathways that regulate lipid and amino acid metabolism and influence their responses. For example, FAO induces M2 phenotypic transformation and inhibiting fatty acid transporter proteins promotes M1 phenotype^161^; M1 macrophages consume glutamine and serine, while producing increased amounts of glycine, which suppresses LPS‐induced M1 polarization in turn.^162^ All of these metabolic adaptations serve to support macrophage activity and maintain their polarization under specific circumstances. However, defining glycolysis as pro‐inflammatory and FAO as anti‐inflammatory may be an oversimplification and there are still many gaps in the research on macrophage metabolic reprogramming in AAA that need to be further explored. Elucidating the mechanisms behind these processes holds the potential to develop new therapeutic strategies for managing this potentially fatal vascular disease. It is essential to further investigate the precise metabolic changes and their functional consequences in AAA‐associated macrophages, which will enhance our comprehension of AAA and provide new avenues for relevant drug discovery and development, ultimately leading to improved clinical outcomes.

## AUTHOR CONTRIBUTIONS

**Ningxin Hou**: Conceptualization; software; visualization; writing—original draft. **Hongmin Zhou**: Formal analysis; validation. **Jun Li**: Formal analysis; validation. **Xiaoxing Xiong**: Investigation. **Hongping Deng**: Investigation; resources; supervision. **Sizheng Xiong**: Conceptualization; project administration; writing—review and editing.

## CONFLICT OF INTEREST STATEMENT

The authors declare no conflict of interest.
